# Advanced Liver-on-a-Chip Model for Evaluating Drug Metabolism and Hepatotoxicity

**DOI:** 10.3390/bios14090435

**Published:** 2024-09-06

**Authors:** Sonia Frojdenfal, Agnieszka Zuchowska

**Affiliations:** Medical Biotechnology, Faculty of Chemistry, Warsaw University of Technology, 00-664 Warszawa, Poland; sonia.frojdenfal.dokt@pw.edu.pl

**Keywords:** liver on a chip, 3D cell model, hydrogel, liver metabolism, drug toxicity

## Abstract

The liver has many important functions, including the biotransformation of drugs and detoxification of the human organism. As such, it is also exposed to many harmful substances, which leads to disorders and diseases such as cirrhosis. For these reasons, it seems important to consider liver metabolism and the direct effects on the liver when evaluating the efficacy of new drugs. Accordingly, we have developed an advanced in vitro liver model using an organ-on-a-chip approach that replicates many of the morphological and functional features of the liver in vivo. The model we created can metabolize drugs, which we demonstrated using two widely used anticancer drugs, 5-fluorouracil (5FU) and capecitabine (CAP). In addition, to the best of our knowledge, we are the first who evaluate the direct effects of these drugs not only on the viability of liver model-building cells but on their functions, such as cytochrome P450 activity and albumin production. Our study brings new hope to properly evaluating drug efficacy at the in vitro level.

## 1. Introduction

The liver is the largest organ of the human body and one of the most important glands responsible for several metabolic functions, including the biotransformation of chemicals [1]. This process can include both the inactivation of substances and their activation into biochemically active variants. Therefore, the consideration of metabolism in evaluating the effectiveness of new therapeutic compounds is extremely important [2]. The liver, through its functions, is also exposed to several harmful factors. A recent review of 111 agents classified as human carcinogens by the International Agency for Research on Cancer (IARC) found that the liver is the second most common chemical-related target site for cancer associated with exogenous exposure [3]. In addition to chemicals, other causes of chronic liver damage include non-alcoholic fatty liver disease (NAFLD) and pathogens [4]. Given the significant role of the liver in physiology, hepatotoxicity is an important determinant of the body’s toxic responses. As a result, drug-induced liver injury (DILI) is a major cause of drug failure in preclinical development, clinical trials, and post-marketing.

Designing and manufacturing drugs is a lengthy process. To make the process more efficient, it is crucial to accurately determine the body’s metabolism and understand the mechanism of a given disease [5]. Most drugs fail in the clinical stages despite their proven efficacy and initial safety in cellular in vitro models and subsequently in animal models. The drug rejection rate in 2021 reached a record high of 95% [6]. The main factors driving this change are the limitations of in vitro models related to the poor reproduction of human body characteristics and animal models, which, despite remaining the gold standard in basic and preclinical research, show vast interspecies differences and a poor prediction of human physiological and pathological states [7]. Therefore, the development of appropriate preclinical models, especially an in vitro liver model that reflects the in vivo conditions of the human liver, is important [8]. A suitable liver model must provide knowledge of the cellular response induced by the influence of the drugs under study, their metabolism, and substances produced in the body. This will enable an early stage, or preclinical studies, to assess the actual activity of the newly developed drug and the extent of any liver damage caused by the drug [9].

Current in vitro cell models, including liver models, are popular due to their simplicity and relatively low cost. However, these simple methods of culturing human cells cause changes in the morphology, physiology, and gene expression of cells, especially hepatocytes, which are the main cells that build the liver. Differences between currently used models and the in vivo environment include the mechanical and chemical properties of the extracellular matrix (ECM), fluid dynamics, and intracellular interactions [10,11]. For these reasons, these cultures cannot sufficiently mimic the structures of tissues and organs; thus, a new trend in biological research is the organ-on-a-chip approach, which, through microtechnology, makes it possible to produce cellular models to faithfully reproduce many properties of the cellular microenvironment by controlling various parameters and design aspects [12]. The advantages of microfluidic cell culture systems include the dynamic flow conditions and mechanical stimulation in their microchannels, mimicking what is observed in vivo. While the main purpose of these models is to simulate the minimal functional units of tissues rather than to create a complete liver tissue, organ-on-a-chip platforms can be used to evaluate the safety and efficacy of various drugs in vitro [12].

Here, we present a novel approach to creating advanced in vitro liver models which replicate many liver features and functions in vivo. Based on our previous studies, in which we showed that triculture liver aggregates provide better natural liver function, we created heterogeneous 3D cell aggregates composed of hepatocytes, stellate cells, and sinusoidal endothelial cells suspended in a hydrogel (mimicking ECM) and cultured in specially designed liver microsystems on a chip to ensure the proper scaling and delivery of essential components to the cells building the 3D aggregates. This approach made it possible to obtain functional liver models, which were confirmed by evaluating the cytochrome P450 activity and urea and albumin production capacity. The study shows that the time of culture and the number of liver aggregates introduced into the microsystem influence the above-mentioned parameters. In addition, the paper demonstrates that the produced liver models can metabolize the selected anticancer drugs, and this metabolism has a significant impact on the effectiveness of breast cancer therapy (MDA-MB 231 cells) with their participation. In addition, we show that the efficiency of drug metabolism depends on the age of the liver model. Moreover, we are among the few to show that the used drug can exhibit hepatotoxicity, not directly affecting cell viability, which is most often assessed, but specific metabolic functions—in our case, albumin production.

## 2. Materials and Methods

### 2.1. Routine Cell Culture

Human hepatocellular carcinoma (HepG2) and human breast adenocarcinoma (MDA-MB 231) cells were obtained from the ATCC Europe Collection. Human stellate (HSCs) cells and human liver-derived endothelial (HSECs) cells were obtained from LONZA (Visp, Switzerland). HepG2 cells were cultured in Dulbecco’s modified Eagle medium (DMEM, Biowest, L0102, Nuaillé, France) with 10% fetal bovine serum (FBS, Biowest, S181B, France), 1% penicillin–streptomycin (Biowest, L0022, France), and 1% L-glutamine (Biowest, X0550). HSCs were cultured in stellate cell growth media (MCST250, LONZA, Visp, Switzerland). HSECs were cultured in Endothelial Cell Growth Medium-2 (EGM-2, PromoCell, C-22011) with 1% penicillin–streptomycin (Biowest, L0022). MDA-MB 231 cells were cultured in Dulbecco’s modified Eagle medium (DMEM, Biowest, L0102) with 10% fetal bovine serum (FBS, Biowest, S181B), 1% penicillin–streptomycin (Biowest, L0022), and 1% L-glutamine (Biowest, X0550). All cell lines were cultured with standard protocols using phosphate-buffered saline (PBS, Sigma-Aldrich, P54931L, USA) and trypsin 0.25%—EDTA (Biowest, L0931, France).

### 2.2. Liver-on-Chip Design and Preparation

The liver-on-chip geometry was designed in AutoCAD^®^ 24.2 The molds for the castings were produced using 3D-printing technology (Hunter DLP 3D printer, Hangzhou, China) in a standard resin FH1100. The printed molds were washed several times with isopropanol and then exposed to UV light for 45 min. After that, the molds were incubated for 24 h at 65 °C.

The molds were filled with liquid degassed poly(dimethylsiloxane) (PDMS) obtained in a ratio of 9:1 (elastomer: crosslinking agent) (SYLGARD™ 184 Silicone Elastomer Kit, Midland, MI, USA) and crosslinked (2 h, 65 °C). The crosslinked PDMS was separated from the molds. Holes were drilled in the designated areas using a Microfluidic PDMS Puncher (Ø 2 mm, Miltex Inc., Plainsboro, NJ, USA). The PDMS parts were washed, dried, and combined with a coverslip using an oxygen plasma generator (Diener, ATTO, Ebhausen, Germany). The microsystems formed in this way were left overnight under load to permanently bond the PDMS layer to the glass surface.

Before starting the 3D culture of liver aggregates in the microsystems, a 2 mg/mL solution of polydopamine in 10 mM Tris HCl (pH 8.5) (Sigma Aldrich, St. Louis, MO, USA) was introduced into the culture chamber and incubated at room temperature for 45 min. The solution was then removed, and the channels were washed with 96% ethanol (Sigma Aldrich, St. Louis, MO, USA). The microsystems were dried with compressed air and stored at 65 °C until use (no more than 3 days).

### 2.3. Diffusion Evaluation in Hydrogel

To evaluate the proper diffusion of nutrients into 3D cell aggregates suspended in hydrogel and cultured in the chamber of the liver-on-chip microsystem, an experiment using fluorescein solution (0.01 mg/mL) was conducted. For this purpose, the fluorescein solution was introduced into the side channels of the microsystem, whose chamber was filled with hydrogel (5 mg/mL collagen type I) without 3D aggregates. Then, images were taken every 20 s using a fluorescence microscope (Olympus IX71, Warsaw, Poland).

### 2.4. Three-Dimensional (3D) Liver Aggregates Formation

Low-adhesion ULA 96-well plates were used to generate 3D liver aggregates. HepG2, HSC, and HSEC cell suspensions were prepared according to standard procedures. Cell density was determined using a cell-counting plate (Fast-Read^®^, Biosigma, Cona (VE) Italy). A cell suspension was prepared at a ratio of 6:3:1 (HepG2:HSEC: HSC), and 10^3^ cells were seeded per well. To minimize cell adhesion to the bottom of the wells in the plate and maximize cell–cell aggregation, the plate was coated the day before with 1% pluronic solution (Sigma Aldrich, USA). Cells seeded in this way were cultured for another 4 days.

### 2.5. Three-Dimensional (3D) Liver Aggregates Transfer to the Liver-on-Chip

Cell aggregates cultured on a low-adhesion ULA 96-well plate were transferred with a pipette along with about 30 µL of a medium into Eppendorf tubes. Preset numbers of aggregates—about 3 or 8, respectively—in each Eppendorf were thus obtained. All aggregates from a given Eppendorf were then taken, resuspended in 5 mg/mL collagen type I, and introduced into the culture chamber in the microsystem. Then, microsystems were incubated for 1 h (37 °C, 5% CO_2_) to crosslink the hydrogel. After this time, the medium formed in a 6:3:1 ratio of DMEM:EGM-2: MCST250 was pipetted into the side channels.

### 2.6. Morphological Changes Evaluation

Observations of aggregate morphology were carried out using a light microscope (Olympus). Images of aggregates were taken at 0, 3, 7, and 10 days of culture. Changes in aggregate size were assessed using ImageJ software 1.8.0 (Ferret diameter and Circularity).

### 2.7. Live/Dead Cell Imaging

Viability testing was carried out by differential staining. Propidium iodide (PI, 1 mg/mL) and calcein–AM (CAM, 5 µg/mL) were prepared at 2 µL and 1 µL per 1 mL of medium, respectively. The medium was withdrawn from the channels, and about 50 µL of the prepared dye solution was introduced and incubated for about 30 min at 37 °C (5% CO_2_). Images were then taken using a fluorescence microscope (Olympus IX71). After MDA-MB 231 cell viability evaluation, the prepared dye solution was introduced to wells (before the drug solution was removed) and incubated for at least 15 min. Images were then taken using a fluorescence microscope (Olympus IX71).

### 2.8. AlamarBlue^®^ Assay

The alamarBlue^®^ (Thermo Fisher Scientific, Waltham, MA, USA) test was used to assess the metabolic activity of MDA-MB-231 cells. The alamarBlue^®^ test contains a fluorometric/colorimetric growth indicator based on the detection of cellular metabolic activity. To perform the test, the medium was removed from the wells and a 10% alamarBlue^®^ solution was added at 100 μL/well. The cells were incubated at 37 °C (5% CO_2_) for 45 min. Fluorescence intensity measurement was made using a CYTATION3 multi-well plate reader (Biokom, Warsaw, Poland) (Em = 552 nm and Ex = 583 nm). Measurements were performed 24 h after incubation with a drug/prodrug that had or did not incubate liver models cultured in liver-on-chip systems.

### 2.9. CellTracker^®^ Labeling

CellTracker^®^ (Sigma Aldrich, USA) solutions were prepared in an FBS-free medium at a concentration of 10 µM. HepG2 cells were stained with CellTracker^®^ Violet, HSC cells with CellTracker^®^ Green, and HSEC cells with CellTracker^®^ Red. The centrifuged cells (3 min, 2000 rpm) were suspended in the prepared CellTracker^®^ solutions and incubated for 30 min (37 °C, 5% CO_2_). After this time, the cells were again centrifuged (3 min, 2000 rpm) and resuspended in 1 mL of fresh medium. Then, suspensions of the three cell types were produced as described above and seeded into wells in a low-adhesion ULA 96-well plate (Corning, Sigma Aldrich, USA). Images were taken after seeding the cells in the wells (day 0) and on days 3 and 7 of culture using a fluorescence microscope (Olympus IX71).

### 2.10. Immunostaining

On days 3 and 10 of culture, some of the aggregates cultured in liver-on-chip microsystems were fixed with 4% formaldehyde. Permeabilization buffer (0.1% Triton™ X-100 in 1X PBS) (Thermo Fisher Scientific, Waltham, MA, USA) was then added to the channels of the microsystems and incubated for 30 min. at room temperature. In the next step, microsystems were washed three times with 1X PBS, and then blocking buffer (1% BSA in 1X PBS) was added and incubated for 1 h at room temperature. After this time, the microsystems were divided into two groups, and the respective primary antibodies (1:100 dilution in blocking buffer for each antibody) were added to each group according to Table S1. Microsystems were incubated overnight in the dark at 4 °C. The next day, microsystems were washed three times with 1× PBS, and then the fluorescent markers Alexa Fluor 488, and Alexa Fluor 657 (both 1:200 dilution in blocking buffer) and NucBlue (1 drop per 1 mL of blocking solution) were added. The microsystems were incubated in the dark for 2 h at room temperature. Then, they were washed three times with 1X PBS, leaving the last portion in the channels. Cell aggregates were imaged using an Olympus FluoView FV10i (Warsaw, Poland) confocal microscope.

### 2.11. Urea Production

To determine the amount of urea produced by cell aggregates, tests were carried out using the Urea Assay Kit (Sigma Aldrich, USA, MAK006). First, a standard solution was prepared by diluting 5 µL of 100 mM Urea Standard Solution in 995 µL of Urea Assay Buffer to obtain a 0.5 mM solution. Then, 50, 48, 46, 44, 42, and 40 µL of Urea Assay Buffer and 0, 2, 4, 6, 8, and 10 µL of the prepared standard were added to a transparent multi-well plate, respectively. Afterwards, 50 µL of each of the media taken from the channels of the microsystem was added to subsequent wells. All wells were supplemented with the reaction mixture according to Table S2. The plate was incubated for 1 h (37 °C, 5% CO_2_). After this time, absorbance was measured using a CYTATION3 plate reader. The amount of urea produced was measured on days 3, 7, and 10 of culture.

### 2.12. Albumin Production

To assess the amount of albumin produced by cell aggregates, the Human Albumin ELISA^®^ Kit (Invitrogen, Carlsbad, CA, USA) was used. To prepare a solution of the albumin standard, 1000 µL of Sample Diluent NS was added to lyophilization, and a final concentration of 100 ng/mL was obtained. A series of dilutions were performed in 10 Eppendorf tubes according to Table S3. The medium from the microsystem channels was taken into Eppendorf tubes and centrifuged (10 min, 2000 rpm). Each medium sample was then diluted 10X in Sample Diluent. Standard and medium solutions were transferred 50 µL to a dedicated 96-well plate, and 50 µL of Antibody Cocktail was added and incubated in the dark on a shaker for 1 h (RT, 400 rpm). After this time, each well was washed three times with wash buffer, and then 100 µL each of CathPoint HRT (Invitrogen, Carlsbad, CA, USA) Development Solution was added and incubated in the dark on a shaker for an additional 10 min (RT, 400 rpm). Fluorescence was measured using a CYTATION3 plate reader (Biokom, Warsaw, Poland). The Human Albumin ELISA^®^ Kit (Invitrogen) was performed on days 3, 7, and 10 of culture.

### 2.13. Cytochrome P450 Activity

The P450-Glo™ Screening Systems (Promega, Madison, WI, USA) assay was used to determine cytochrome P450 CYP3A4 activity. The medium was aspirated from the channels of the microsystems, and 60 µL each of luciferin (LUC-IPA) prepared in culture medium (1 µL LUC-IPA per 1 mL medium) was added and incubated for 1 h (37 °C, 5% CO_2_) in the dark. Then, 50 µL of medium was taken from each microsystem and transferred to a new white multi-well plate. Afterwards, 50 µL of luciferin detection reagent was added to each well and incubated at room temperature for 20 min isolated from light. After this time, luminescence was measured using a CYTATION3 (Biokom, Warsaw, Poland) plate reader. The test was performed on days 3, 7, and 10 of culture.

### 2.14. Drug Metabolism Evaluation

First, 5-fluorouracil (5FU) and its prodrug capecitabine (CAP) at a concentration of 100 µM were introduced into microsystems with liver models (day 7 of culture) and incubated for 24 h. After this time, the carrier along with the metabolized drugs was collected from the channels and transferred to multi-well plates on which MDA-MB 231 cells were seeded the day before (10^4^ cells/well). In some microsystems, differential staining, analysis of cytochrome P450 activity, and albumin production were performed; in others, the media was changed to fresh and continued culture. The activities were repeated on the 10th day of culturing liver models in liver-on-chip microsystems. MDA-MB 231 cells, after 24 h of incubation with metabolized drugs (or in free form—positive control), were analyzed using the alamarBlue^®^ (Thermo Fisher Scientific, Waltham, MA, USA) test and differential staining.

### 2.15. Statistical Analysis

Data were expressed as the mean ± standard deviation. Origin 2021b software was used for statistical analysis. *p* values for statistical significance were obtained by using an analysis of variance test (Tukey’s multiple comparisons). *p* < 0.05 was considered significant. The data are representative of at least 3 independent experiments.

## 3. Results

### 3.1. Liver-on-Chip Design Optimization

**Figure 1:**
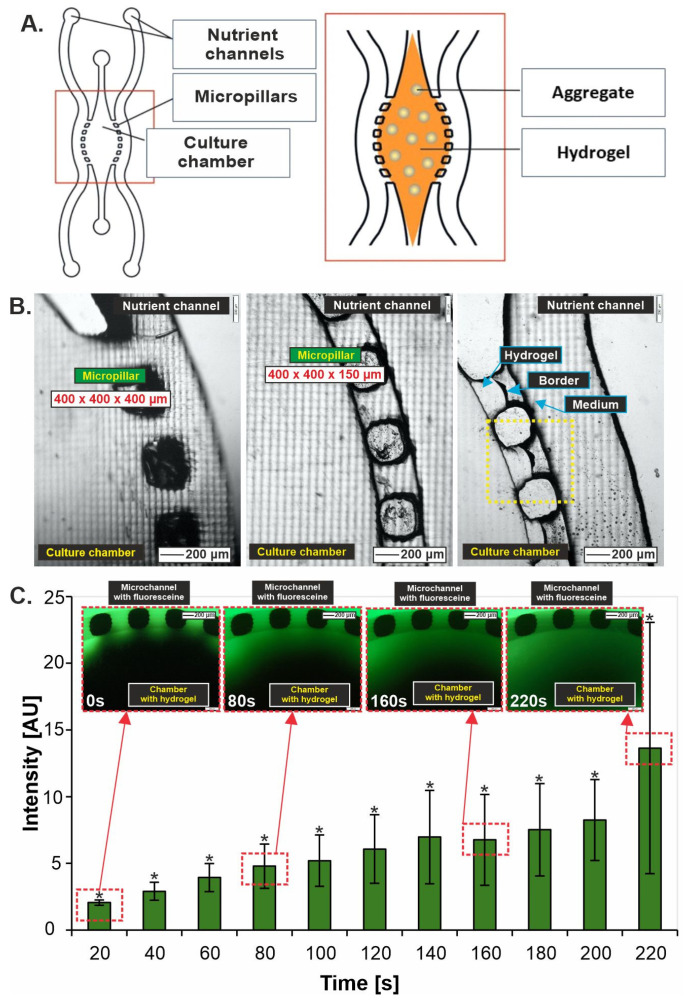
Liver-on-chip characterization. (**A**) Liver-on-chip geometry with a schematic representation of a culture of liver aggregates suspended in a hydrogel. (**B**) Optimizing the size of the pillars between the culture chamber and the culture media supply channels. (**C**) Simulation of the rate of nutrient diffusion into aggregates suspended in hydrogel and placed in a culture chamber in a liver-on-chip microsystem, N = 3, *p* < 0.05. “*” indicate *p* < 0.05.

The designed microsystem is shown in Figure 1A. In the center of the microsystem was the culture chamber, which was separated from the side channels supplying the medium by micropillars. The culture chamber was provided with an inlet and outlet channel, which allowed the introduction of liver aggregates suspended in the hydrogel. Initially, three versions of microsystems were proposed, differing in the dimensions of the micropillars, namely 400 × 400 × 400 μm, 300 × 300 × 400 μm and 400 × 400 × 150 μm (length × width × height). In the case of the former (400 × 400 × 400 μm) and the latter (300 × 300 × 400 μm), micropillars were damaged during the separation of the PDMS layer from the printed mold (Figure 1B). The consequence of this at a later stage during the introduction of the hydrogel was that it leaked from the culture chamber space into the side channels. The third microsystem with 400 × 400 × 150 μm saws allowed the PDMS to be freely separated from the printed mold without damaging the micropillars (Figure 1B) and allowed the hydrogel to be retained in the culture chamber (Figure 1B). Therefore, this version of the microsystem was chosen for further studies.

Before proceeding with bioassays, it was ascertained that the diffusion of the culture medium and therefore of nutrients into liver aggregates suspended in the hydrogel was adequate. Accordingly, an experiment was conducted using fluorescein solution (0.01 mg/mL). Collagen type I (5 mg/mL) without suspended aggregates was introduced into the culture chamber, crosslinked, and then fluorescein solution was introduced into the side channels, and its permeation was observed. Images were taken every 20 s. It was observed that diffusion into the depths of the hydrogel-filled culture chamber occurred smoothly and in a short period of time. After about 5 min, complete filling of the chamber was observed (Figure 1C).

### 3.2. Morphological Characterization of 3D Liver Models

**Figure 2:**
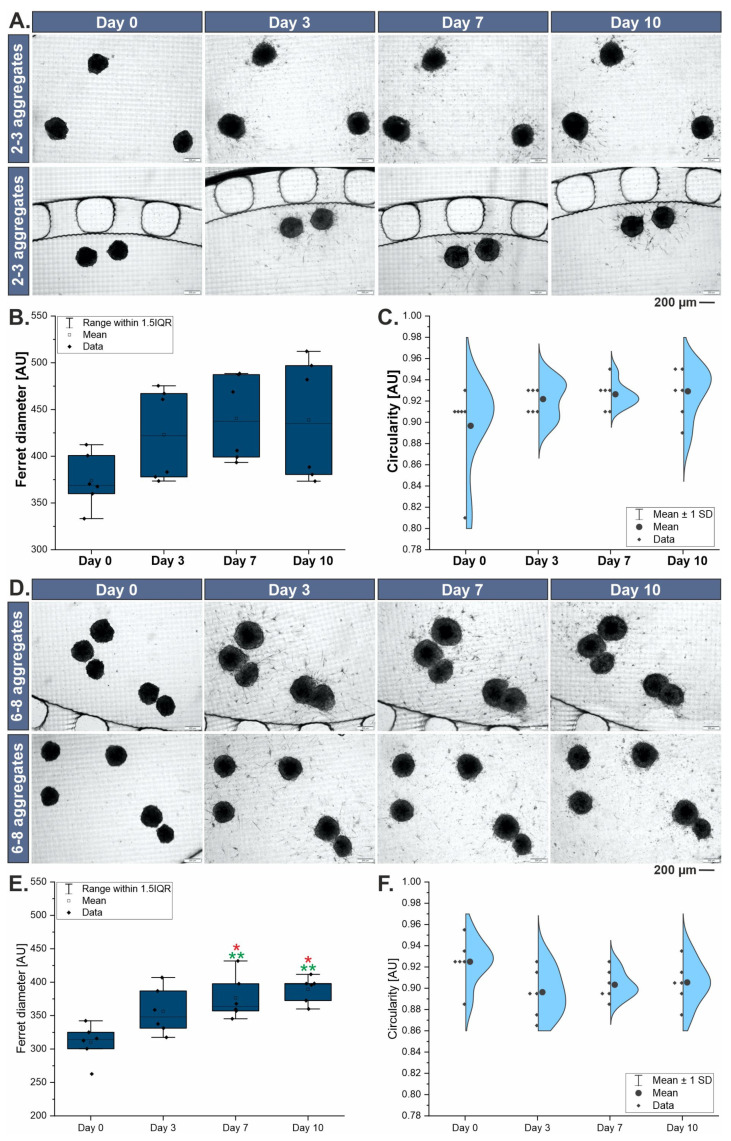
Changes of liver aggregates in liver-on-chip microsystem. (**A**) Morphology of aggregates cultured in the hydrogel in liver-on-chip (2–3 aggregates per chamber). (**B**) Changes in Ferret diameter of liver aggregates during 10-day culture (2–3 aggregates per chamber). (**C**) Changes in circularity of liver aggregates during 10-day culture (2–3 aggregates per chamber). (**D**) Morphology of aggregates cultured in the hydrogel in liver-on-chip (6–8 aggregates per chamber). (**E**) Changes in Ferret diameter of liver aggregates during 10-day culture (6–8 aggregates per chamber). (**F**) Changes in circularity of liver aggregates during 10-day culture (6–8 aggregates per chamber). The red “*” indicate *p* < 0.05. The green “**” indicate *p* < 0.01.

**Figure 3:**
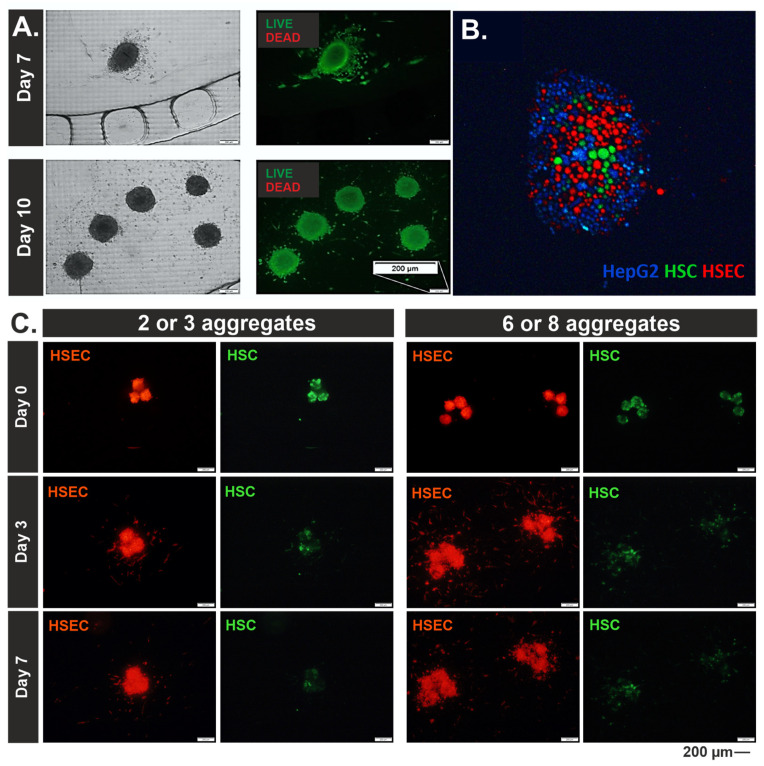
Analysis of cell migration from the aggregate structure to the hydrogel space. (**A**) Viability staining with calcein AM (CAM) and propidium iodide (PI) after 7 and 10 days of culture. (**B**) Cell labeled with three different CellTracker^®^ just after cell seeding in ULA plate. (**C**) Analysis of HSEC and HSC location during the aggregates culture in liver-on-chip.

About three or eight 3D liver aggregates were introduced to the systems. Manually introducing a specific number of aggregates to the system was quite difficult. However, due to the repeatability of the results, they were divided into two groups with the number of aggregates specified in the following ranges: two to three aggregates and six to eight aggregates. Observations of the change in morphology and size of cell aggregates were carried out at 0, 3, 7, and 10 days of culture (Figure 2). Qualitative (images) and quantitative (change in Ferret diameter and circularity) analysis was conducted. It was evaluated how the culture time and the number of liver aggregates present in the culture chamber affect these parameters. It was noted that the aggregates increased in size with the time of culture in the microsystem, which may be indicative of the cell proliferation taking place (Figure 2B,E). The progression of increasing aggregate diameter/size was dependent on the number of aggregates in the culture chamber. The size of aggregates in chambers with more aggregates grew more slowly than in chambers with fewer aggregates. This may be a result of cellular interactions and space limitations for growth. The circularity of the aggregates in each case studied was preserved, throughout the culture, and averaged ~0.9 (Figure 2C,F). In addition, using differential staining, it was shown that the cell-building liver aggregates cultured in the microsystem had high viability in the 10-day culture draught (Figure 3A), which is also a confirmation of sufficient diffusion of nutrients to all 3D aggregate-building cells.

During culture in the microsystem, the migration of single, fibrous cells outside the 3D structures of the aggregates was observed (Figure 2 and Figure 3A). This effect was evident as early as day 3 of culture and was more intense when the aggregates were close to each other. This could likely be related to communication between cell aggregates placed in a single microsystem. However, to determine which cell type migrates outside the aggregate, additional tests were performed. For this purpose, cells of each type were labeled with a different CellTracker^®^ and seeded on ULA plates (Figure 3B). Thus, HepG2 cells showed blue fluorescence, HSEC cells showed red fluorescence, and HSC cells showed green fluorescence. At the same time, this test allowed us to confirm that the ratio of each HepG2:HSEC: HSC cell type corresponded to the assumed ratio, i.e., 6:3:1. After the aggregates were formed, they were transferred to chambers in microsystems as described above. Photographs were taken on days 3 and 7 of culture.

Unfortunately, as early as day 3 of the culture, blue fluorescence (HepG2 cells) was not observable. Perhaps this is due to the rapid divisions of HepG2 cells and thus the reduction in the amount of dye contained in the cytoplasm. Despite this, fluorescence observation of the remaining cells, green—HSC and red—HSEC, was visible. It was noted that the cells migrating out of the aggregate area were HSEC cells (Figure 3C). Thus, it can be thought that they are involved in the transmission of signals between aggregates within a given microsystem. In the in vivo environment of the liver, sinusoidal cells arrange themselves to form tubular structures that allow the transport of substances from the blood to the hepatocytes and vice versa. In addition, endothelial cells form blood vessels in the liver, which, in the case described here, may indicate the initiation of formation through the intercellular signaling of vasculature. Therefore, an attempt may be made in the future to create a vascular network around 3D aggregates.

**Figure 4:**
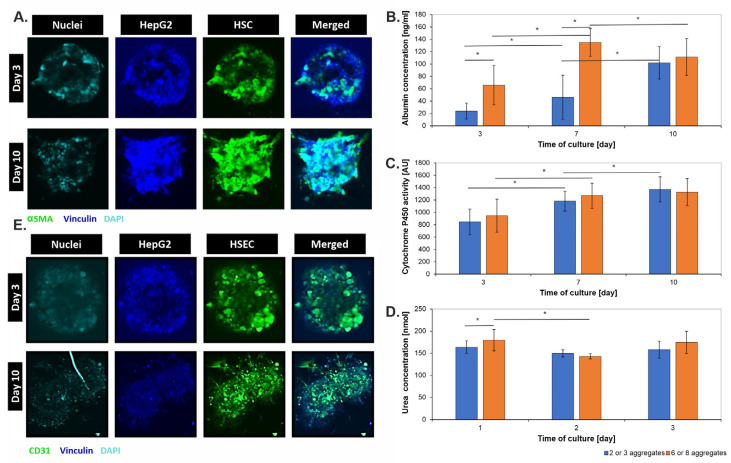
Change in the amount of albumin (**A**), activity of cytochrome p450 (**B**), amount of urea (**C**) in the liver-on-chip microsystems depending on the number of aggregates and the time of culture. (**D**,**E**) Cell location in the aggregate structure during 10-day culture in liver-on-chip microsystems. Immunostaining with αSMA (HSC cells), Vinculin (HepG2 cells), and CD-31 (HSEC cells). “*” indicate *p* < 0.05.

Since CellTracker^®^ staining was not stable over an extended culture period (10 days), to analyze the distribution of each cell type in a 3D liver aggregate suspended in hydrogel and cultured in a microsystem, it was decided to perform staining using antibodies (Figure 4A). For this purpose, three types of antibodies specific to each cell type were selected. Vinculin was selected for HepG2, CD31 protein was selected for HSEC, and αSMA protein was selected for HSC. Staining was performed on days 3 and 10 of culture. The different solutions necessary to economize subsequent stages of immunostaining were introduced into the side channels. Observing the changes between the 3rd and 10th days of culture, the proliferation of aggregates and the formation of irregular shapes by them is evident. This confirms the observations mentioned earlier about HSEC cells migrating to form an extracellular matrix and cells contacting each other between distant aggregates. In addition, HepG2 cells in both groups occupy the most space at the beginning of the culture and are located at the periphery of the clump. As the cells grow and proliferate, more and more space begins to be occupied by HSC and HSEC cells. However, the obtained images do not allow a full analysis of the distribution of cells in the 3D structure. The immunostaining procedure would likely have to be adapted to the hindered conditions of diffusion due to (i) microtechnology, (ii) hydrogel, and (iii) 3D cell model. In the future, the method should be modified for more efficient results.

### 3.3. Metabolic Characterization of 3D Liver Models

The models produced in the microsystems were characterized in terms of liver-specific metabolic functions, namely urea production, albumin production, and cytochrome P450 activity. The studies were conducted on days 3, 7, and 10 of culture for two types of culture: (i) microsystems containing two to three liver aggregates and (ii) microsystems containing six to eight liver aggregates (Figure 4). In conducting the study, it was assumed that with the number of aggregates in the culture and with the time of culture (more cells—proliferation), the amount of urea, the amount of albumin, and the cytochrome p450 activity should increase.

The albumin concentration increased with culture time and the number of aggregates in the microsystem (Figure 4B). In microsystems containing two to three aggregates, increasing albumin concentration has no limiting effect on further albumin production (with time). However, the graph shows a decrease in concentration for day 10 in the case of a microsystem containing six to eight aggregates. Perhaps, as with urea, once a certain concentration is reached, it has a limiting effect on the cells by which they do not produce more albumin. To see what threshold concentration affects the reduction in albumin production by liver cells, one would have to run aggregated cultures for a longer period of time. Another aspect of the decrease in albumin production for day 10 of culture could be the reduction in albumin secretion outside the aggregates. A larger number of aggregates yields more stored albumin, causing the result to drop significantly during measurements.

Cytochrome p450 activity increases with culture time (Figure 4C). Statistically significant differences were noted between days 3, 7, and 10 for microsystems with two to three aggregates. The results show an increasing activity of the cytochrome, which excludes the formation of toxic metabolites of the reactions taking place in the cells. In the case of microsystems with more aggregates (six to eight aggregates), the differences in results between the 7th and 10th days of culture are not significantly different, and considering the standard deviation, they are close to each other. Also, in this case, there is an inhibition of the studied value. It can be thought that this is due to the excessive number of cell aggregates in the microsystem, so that the compounds produced during culture have a limiting effect on cell growth and proliferation and thus on the activity of cytochrome p450.

The amount of urea produced remains constant throughout the culture for both experimental microsystems (Figure 4D). A slight decrease, sustained over the following days, was recorded for the 7th day of culture, which may be due to the aggregation of cells that takes place, and their tighter packing leads to restrictions in the secretion of the substance outside the aggregate. No significant differences in the amount of urea produced were noted between microsystems with different numbers of aggregates. Perhaps the amount of urea produced does not depend on the number of cells, or there is a threshold concentration where cells inhibit production once a certain level is reached. However, this hypothesis would need to be confirmed in other studies.

### 3.4. Drug Metabolism Studies

**Figure 5:**
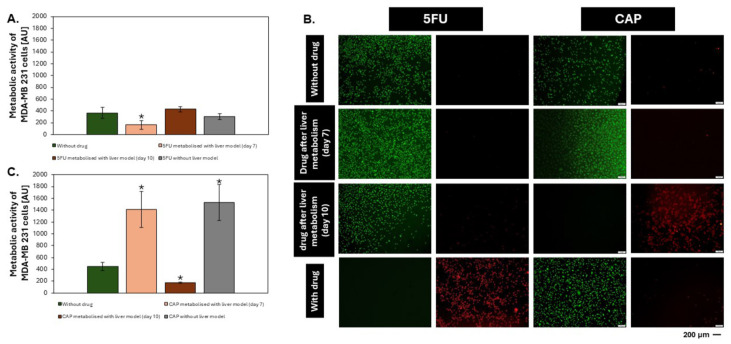
Studies of the metabolic ability of liver models to biotransformed anticancer drugs in two forms—as a pure drug and as a pro-drug. (**A**,**C**) Metabolic activity/viability of MDA-MB-231 cells—alamarBlue^®^ assay. (**B**) Images of MDA-MB-231 cells, differential staining (green—live cells; red—dead cells). “*” indicate *p* < 0.05.

The metabolic activity of MDA-MB-231 cells after treatment with 5FU in its pure form and after hepatic metabolism by the liver-on-chip model cultured for 7 and 10 days did not differ significantly from the control (Figure 5A). However, the results of differential staining indicate a stronger effect of the drug after direct administration to MDA-MB-231 cells than in the case of transfer of the medium with this substance after 24 h of incubation with liver cells (Figure 5B). The observed results suggest that 5FU administered to the liver is broken down into an inactive substance, the effect of which does not produce the same effect as in the case of the direct administration of 5FU to MDA-MB-231 cells. The liver in the human body is responsible for deactivating toxic ingredients into their inactive form, which can be excreted by the body, reducing its side effects. In this case, it can be assumed that 5FU is assessed by liver cells as a harmful substance, and its decomposed form does not have the same effect.

In the case of MDA-MB 231 cells treated with CAP in its pure form and metabolized by the liver (liver model on days 7 and 10), it was noticed that their metabolic activity varied significantly depending on the degree of drug metabolism. It was noticed that there was no effect of CAP without incubation with liver cells on the MDA-MB 231 line (Figure 5C). According to microscopic images, breast cancer cells also showed no reaction to the administered prodrug administered in its pure form (Figure 5B). This is a positive result of the CAP test, which, as a prodrug, should not affect cells until it is properly metabolized in the body to its active form. Nevertheless, as can be seen in the graph, CAP did not show any effect after incubation with the liver models from day 7, which may indicate that the metabolic activity of the liver models on this day was too low. However, CAP metabolized by the day 10 liver models performed its function by reducing the metabolic activity of MDA-MB 231 cells, leading to their death. The results are consistent with the differential staining results (Figure 5B).

Microscopic photos of liver aggregates from day 7 and 10 of culture after 24 h of incubation with 5FU and CAP show that the cells building the aggregates remained alive, which suggests that neither 5FU nor CAP is toxic to the cells building the prepared liver models. Additionally, living cells can be observed in the hydrogel around the aggregates, which, as confirmed above, indicates the presence of hepatic sinusoidal endothelial cells (HSEC), which are responsible for the contact of the aggregates with the environment.

**Figure 6:**
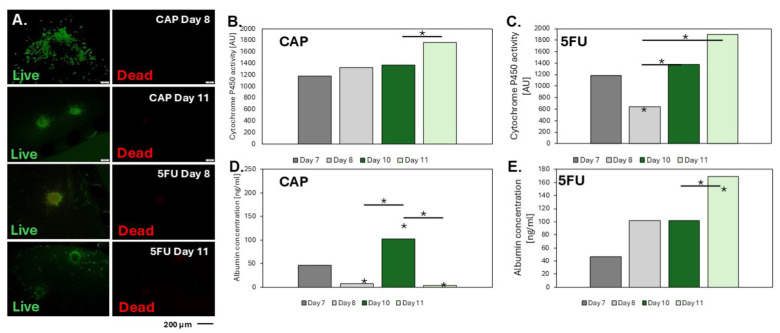
Effect of tested drugs on liver models. (**A**) Viability of 3D aggregates in microsystems after administration of test compounds. (**B**,**C**) Change in cytochrome P450 activity after contact of liver models with the CAP and 5FU, respectively. (**D**,**E**) Albumin production depends on the culture time and the type of compound administered—CAP and 5FU, respectively. “*” indicate *p* < 0.05.

An increase in cytochrome activity during the culture was observed for liver aggregates treated with the CAP (Figure 6B). The response of the models was like the control (Figure 4B). This may indicate the lack of formation of toxic metabolites of reactions occurring in cells and the lack of influence of CAP on the tested parameter. Looking at the results of cytochrome P450 activity for aggregates administered with the 5FU drug, the inhibition of the tested value only for aggregates from the 7th day of culture (Figure 6C) was observed. It can be assumed that when the drug is metabolized by a model that may not be stabilized yet, compounds are produced that limit cell development and proliferation, which reflects a decrease in cytochrome activity. However, it was noticed that depending on the compound used (CAP or 5FU), albumin production was completely different (Figure 6D,E). After administration of the prodrug (for days 7 and 10), albumin production decreases, while after the administration of 5FU, the upward trend is maintained throughout the culture (as for the control Figure 4A). The presented results may indicate a negative impact of CAP on albumin production and thus damage to one of the basic liver functions. The reason may be that albumin production is impaired by increased CAP metabolism. However, additional studies would need to be conducted to prove this supposition.

## 4. Discussion

The conducted research aimed to optimize the fabrication of a 3D model of the liver in organ-on-chip microsystems in vitro and to evaluate the possibility of using the fabricated model to study the metabolism of anticancer drugs. During the work, the focus was on the design and fabrication of a liver-on-chip microsystem allowing the efficient and simple establishment of cell aggregate cultures in hydrogel. Tests were planned to verify whether liver cells retain specific functions in the proposed microsystem and whether the measured parameters of these functions increase or remain constant with the time of culture. In addition, as a proof of concept, studies on the ability to metabolize selected anticancer drugs (5-fluorouracil and its pro-drug capecitabine) by microsystem-generated advanced liver models and the direct effects of these drugs on liver models (possible hepatotoxicity) were conducted.

Based on the results, it was concluded that the culture of aggregates in the hydrogel in a microsystem highly reflects the situation in vivo. The effect of the number of hepatic aggregates introduced into the microsystem (two to three or six to eight aggregates) and the time of culture on selected characteristic functions performed by the liver in vivo was evaluated. According to the hypothesis, which assumed that with the time of culture, urea and albumin production and cytochrome activity would increase, the conditions were mostly met by the microsystem containing two to three aggregates [13,14]. It can be thought that such several cells contained in the microsystem do not create limitations to produce the tested substances and do not generate a toxic environment. In the case of urea production, no increment was noticed over time, the liver models in this case showed a more even level of the amount of urea produced. In addition, this study showed that compared to culture under standard conditions, the efficiency of urea production at the microscale is higher [15]. To more accurately assess the progress of urea production over time, such a culture would need to be extended. This would make it possible to visualize whether the level of production will oscillate around similar values, or whether, however, the production will increase or decrease.

The methodology developed in this work for generating and culturing liver models stands out from previous published work [16,17,18,19]. First, in this study, the co-culture of cells of three lineages that make up the liver in vivo (hepatocytes, stellate cells, sinusoidal endothelial cells) was used to produce 3D aggregates, which undoubtedly brings the created model closer to the natural structure of this organ. In addition, in this work, the 3D-generated liver aggregates were suspended in a hydrogel, the purpose of which was to simulate the extracellular matrix. Moreover, the research was conducted in a specially designed microsystem, the dimensions of which ensured the correct volume-to-volume (SAV) ratio, which makes the results obtained with this type of model correspond to the situation in the living organism to a more considerable extent than standard approaches. Jang et al. used only cell culture of the HepG2 line in their experiment, which nevertheless allowed them to show that a microscale culture allows for several times higher ratios for specific liver functions than for standard cultures [20]. However, such a model, despite promising results, does not sufficiently reflect in vivo conditions of the lack of presence of other liver-building cells, which, as we know, in addition to the specific functions they perform, also stimulate each other and allow for proper functioning. This is especially the case when culturing hepatocytes, where it has been proven that the presence of other cells like sinusoidal cells, stellate cells, or Kupffer cells, allows for a more efficient production of urea or albumin [9]. Therefore, the development of a liver model should no longer be based on the use of only one cell line. It is necessary to construct such a model that is multicultural of liver-building cells, just as it is in the human body. Creating a model in which we have juxtaposed three liver-building cell lines brings us closer to obtaining reliable results that are adequate for in vivo conditions. In this study, we used hepatocytes, which perform ATP-requiring tasks such as albumin and glycogen synthesis, participate in gluconeogenesis or the urea cycle, contribute to ammonia metabolism, and are involved in various cytochrome P450-dependent metabolic reactions [21]. In addition, the study used human hepatic sinusoidal endothelial cells (HSECs)—which are highly specialized endothelial cells that form a permeable barrier between blood cells on the one hand and hepatocytes and hepatic stellate cells on the other. They are not only a physical barrier but contribute to various physiological and pathological processes, including metabolite transport, inflammation, angiogenesis, and vascular tone [22]. In addition, hepatic stellate cells (HSCs) are essential for both hepatocytic function and lesions, which are hallmarks of liver fibrogenesis and carcinogenesis. HSCs can influence the growth, differentiation, or morphogenesis of all liver parenchymal cells, as evidenced by the close contact during development between stellate cells, endothelial cells, hematopoietic cells, and liver epithelial cells. In addition to their implications in liver injury, HSCs play a key role in liver development and regeneration, xenobiotic response, intermediary metabolism, and regulation of the immune response [23]. Ho et al., taking up the challenge of creating cocultures of HepG2 and HUVECs despite achieving the structure of the hepatic stroma and using flow culture, failed to replicate the 3D structure of the liver. The model they use is interlaced cells of two lines adjacent to a substrate (2D culture) [24]. The 2D culture is not a reflection of the real state, where cells grow in 3D and are otherwise surrounded by extracellular matrix. However, it has been proven that most hepatocytes cultured in 2D lose their intrinsic biochemical signals and the cell–cell communication necessary to maintain a physiological phenotype and cannot fully reproduce liver-specific functions [25]. In addition, many studies have shown that culturing hepatocytes in a 3D structure not only mimics liver architecture but also improves cell–cell and cell–extracellular matrix interactions and supports internal liver functions [16]. In addition, 3D liver models are characterized by long-term genetic stability, in vivo-like organization, and the maintenance of essential cellular crosstalk [26]. Biomimetic perfusion culture (3D-LOC) used only hepatocytes to generate 3D liver models [27]. The authors used an uncontrolled separation of liver cells into U-shaped wells. However, this method poses the risk of forming 3D aggregates of heterogeneous shapes and sizes and can lead to the formation of a necrotic core if there are too many cells per well. In contrast, the controlled method we developed for establishing cell aggregate cultures in which no necrotic core is present offers a chance to obtain reliable results. The work of Lee et al., which used cells of the HepG2 and HSC lines, shows in detail the interaction between the cells of these two lines [16]. The flow, generated in the developed chip, is crucial when obtaining results because it is with the medium that signal molecules are transferred between the culture chambers from HSC cells to HepG2. Nevertheless, in this experiment as well, the researchers combined two culture chambers containing a monolayer of HSC cells with three-dimensional aggregates of HepG2 cells, which were also formed in an irregular fashion. Such a study indicates the existence of cell–cell relationships but does not approximate the conditions created and the type of culture to the in vivo condition. Juxtaposing the results obtained in this study with literature reports, the designed microsystem meets many of the conditions necessary to obtain reliable experimental results. First, it allows obtaining 3D cell aggregates of three liver-specific lines in a controlled manner, in which no necrotic core is observed during the 10-day culture, and using hydrogel, an environment mimicking the ECM matrix is provided. When planning further studies, it would be necessary to check the specific functions of liver cells during the application of flow conditions. In the described experiment, the medium was exchanged on each of the days of the experiments conducted with a pipette, so the culture was conducted under static conditions. The use of flow would allow another approximation to mimic the natural in vivo environment prevailing in the liver during blood flow. The results obtained in the above work made it possible to prepare a microsystem with a certain number of cell aggregates, which, despite being cultured under static conditions, showed better hepatic function than the static cultures we had previously conducted in a 96-well plate [15].

Synthesizing suitable substances that have a therapeutic effect on the body, which will fulfill several properties and preserve the body’s natural functions, is very difficult. As shown in the above experiment, substances whether in active or inactive form can exhibit both desirable actions and harmful effects in specific areas. Given the significant role of the liver in physiology, hepatotoxicity is an important determinant of the body’s toxic responses. As a result, drug-induced liver damage is a major cause of drug failure in preclinical development, clinical trials, and post-pharmaceutical marketing. In our study, we showed that a properly designed and fabricated liver-on-chip model is capable of metabolizing drugs both in free form and as a prodrug. A drug that encountered the liver model exhibited different therapeutic properties than a drug that was directly administered to cancer cells, and for this reason, it is important to take liver metabolism into account when testing new drugs in vitro. In addition, we have shown that drugs can have detrimental effects on the liver model itself—affecting its characteristic functions. This result is also extremely important for the in vitro testing of new drugs. Sometimes, side effects can have a more negative impact on the human body than the disease which is treated.

## 5. Conclusions

The research conducted allowed optimizing the fabrication of a 3D liver model based on the organ-on-chip approach. The presented work is one of the few that considers such several important in vivo features as heterogeneity, the presence of ECM, appropriate scaling, and three-dimensionality. In this work, we showed that the in vitro liver models we produced, perform important and characteristic functions of the liver in vivo, such as urea production, albumin production, and high cytochrome P450 activity. In addition, we showed that the models we produced are capable of efficiently metabolizing selected anticancer drugs. We were the first to show that the efficiency of drug metabolism depends on the length of the culture of liver models. Moreover, in addition to the direct effect of liver metabolism on the efficacy of anticancer therapy, we were one of the few to show that a drug can also have a significant direct effect on a liver model. We have shown that despite the lack of effect of drugs on liver cell viability, these drugs can affect the functions they perform, as in the case of the inhibitory effect of CAP on albumin production shown in our study. The model presented in this paper, as well as the results obtained using it, can provide a basis for further research in the field of new therapies, in vitro cellular models, and biotechnology.

## Data Availability

The raw data supporting the conclusions of this article will be made available by the authors on request.

## Supplementary Materials

The following supporting information can be downloaded at: https://www.mdpi.com/article/10.3390/bios14090435/s1, Table S1: Used antibodies depending on the group; Table S2: Composition of the reaction mixture; Table S3: Dilutions of albumin standard.
