# Supplementary material for: Advanced Liver-on-a-Chip Model for Evaluating Drug Metabolism and Hepatotoxicity
Source: Biosensors (Basel). 2024 Sep 6;14(9):435. doi: 10.3390/bios14090435 (PMC11430604; doi:10.3390/bios14090435)
Supplement: Supplementary file 1 [file biosensors-14-00435-s001.zip › biosensors-3141724-supplementary.pdf]

Advanced Liver-on-a-Chip model for evaluation of the degree of drug metabolism and their hepatotoxicity

Sonia Frojdenfal[1], Agnieszka Zuchowska[1]*

[1]Chair of Medical Biotechnology, Faculty of Chemistry, Warsaw University of Technology, Poland

*agnieszka.zuchowska@pw.edu.pl

Table S1. *Used antibodies depending on the group.*

**Group 1**	Vinculin Rabbit – specific for HepG2
	αSMA Mouse – specific for HSC
**Group 2**	N-Cadherin Mouse – specific for HepG2
	CD31 Rabbit – specific for HSEC

Table S2. *Composition of the reaction mixture.*

**Substance/Reagent**	**Volume [µl]**
Urea Assay Buffer	42
Peroxidase	2
Enzyme Mix	2
Developer	2
Converting Enzyme	2

Table S3. *Dilutions of albumin standard.*

**Sample number**	**Volume of standard [µl]**	**Volume of NS [µl]**
Standard 0	300; standard 100 ng/ml	0
Standard 1	150; Standard 0	150
Standard 2	150; Standard 1	150
Standard 3	150; Standard 2	150
Standard 4	150; Standard 3	150
Standard 5	150; Standard 4	150
Standard 6	150; Standard 5	150
Standard 7	150; Standard 6	150
Standard 8	150; Standard 7	150
Standard 9	150; Standard 8	150
